# S3 Guideline for the treatment of psoriasis vulgaris, adapted from EuroGuiDerm – part 1: Treatment recommendations and monitoring

**DOI:** 10.1111/ddg.16002

**Published:** 2026-01-14

**Authors:** Alexander Nast, Andreas Altenburg, Matthias Augustin, Frank Bachmann, Wolf‐Henning Boehncke, Markus Cornberg, Hilte Geerdes‐Fenge, Brit Häcker, Peter Härle, Joachim Klaus, Michaela Köhm, Arno Köllner, Ulrich Mrowietz, Hans‐Michael Ockenfels, Antonia Pennitz, Sandra Philipp, Thomas Richter, Thomas Rosenbach, Tom Schaberg, Martin Schlaeger, Gerhard Schmid‐Ott, Michael Sebastian, Karisa Thölken, Ralph von Kiedrowski, Uwe Willuhn, Christoph Zeyen

**Affiliations:** ^1^ Division of Evidence‐Based Medicine (dEBM) Department of Dermatology Venereology and Allergology Charité – Universitätsmedizin Berlin corporate member of Freie Universität Berlin and Humboldt‐Universität zu Berlin Berlin Germany; ^2^ Department of Dermatology Venereology and Allergology Dessau Municipal Hospital Dessau Germany; ^3^ Institute for Health Services Research in Dermatology and Nursing (IVDP) University Medical Center Hamburg‐Eppendorf (UKE) Hamburg Germany; ^4^ Dermatology Center Berlin Siddi & Bachmann Berlin Germany; ^5^ Service de Dermatologie et Vénéréologie Hôpitaux Universitaires de Genève Geneva Switzerland; ^6^ Department of Gastroenterology Hepatology and Endocrinology Hannover Medical School Hannover Germany; ^7^ Department of Internal Medicine, Tropical Medicine and Infectious Diseases Rostock University Medical Center Rostock Germany; ^8^ German Central Committee against Tuberculosis (DZK) Berlin Germany; ^9^ Rheumatology Center in conformity with G‐BA Department of Rheumatology Clinical Immunology and Physical Therapy Marienhaus Hospital Mainz Mainz Germany; ^10^ German Psoriasis Federation Hamburg Germany; ^11^ Division of Rheumatology Immunology – Inflammation Medicine University Hospital Frankfurt Goethe University Frankfurt am Main Germany; ^12^ Fraunhofer Institute for Translational Medicine and Pharmacology ITMP Frankfurt Frankfurt am Main Germany; ^13^ Dermatology Office Duisburg Germany; ^14^ Psoriasis Center Department of Dermatology Venereology Allergology University Hospital Schleswig‐Holstein Campus Kiel Kiel Germany; ^15^ Department of Dermatology and Allergy Hospital Hanau Hanau Germany; ^16^ Dermatology Office Oranienburg Germany; ^17^ Helios Versorgungszentren GmbH MVZ Gotha Gotha Germany; ^18^ Dermatology Office Osnabrück Germany; ^19^ Dermatology Office Oldenburg Germany; ^20^ Berolina Hospital Löhne Germany; ^21^ Dermatology Office Mahlow Germany; ^22^ Department of Dermatology and Allergology University Hospital Augsburg Augsburg Germany; ^23^ Dermatology Office Selters Germany

**Keywords:** Monitoring, psoriasis, recommendations, scores, targets

## Abstract

This first part of the updated German S3 guideline on the treatment of psoriasis vulgaris covers the sections on treatment recommendations, treatment goals, and monitoring of therapies. The recommendations are based on the current Cochrane network meta‐analysis, the results of which are also summarized. When selecting systemic therapies for psoriasis vulgaris, the guideline emphasizes consideration of efficacy, safety, comorbidities, and individual patient factors. The decision framework is presented in the treatment options overview, and in this updated version, the possibility of first‐line therapy with biologics or novel targeted small molecules is more prominently highlighted. Standardized instruments for assessing disease severity, as well as patient‐centered treatment goals, are underscored. A Psoriasis Area and Severity Index (PASI) 75 response is defined as the minimum therapeutic target, while PASI 90 or an absolute PASI <2 are considered desirable goals. Since the last version, two newly approved agents, bimekizumab and deucravacitinib, have been incorporated, accompanied by specific usage recommendations. Among the established therapies, guidance on methotrexate has been extensively revised, particularly regarding administration, dosing, and monitoring. This guideline aims to provide clinicians with an evidence‐based framework for therapy selection and monitoring, while strengthening shared decision‐making with patients.

## NOTES ON USING THE GUIDELINE

This publication includes selected text passages where particularly relevant modifications have been made. Apart from these sections presented in part 1, part 2 includes the “Guidance for specific clinical and comorbid situations”.

The long version of the guideline is available on the AWMF pages (https://register.awmf.org/de/leitlinien/detail/013‐001). Particular attention should be paid to the information provided in the chapter “Notes on using the guideline/Disclaimer” in the long version when applying the guideline recommendations presented in this short version. The following accompanying documents of version 8 are available on the AWMF pages: Appendix A (“Recommendations for topical therapy”, “Phototherapy”, “Other therapies”, “Interface definition”), Evidence Report, Guideline Development Report with information about conflicts of interest and a set of PowerPoint slides on guideline implementation.

## WHAT IS NEW?

The sections on disease severity and treatment goals, general recommendations for initiating and selecting a systemic therapy, overview of treatment options and the section presenting the efficacy and safety of therapies have been revised based on the Cochrane network meta‐analysis. Bimekizumab and deucravacitinib have been incorporated in the guideline including corresponding notes on performing the therapy. The content of the chapter on methotrexate has been revised. In the section “Guidance for specific clinical and comorbid situations”, the chapters “Screening for tuberculosis”, “Management of psoriasis in patients with latent tuberculosis” and “Viral hepatitis” have been extensively revised. In addition, the recommendations for “Systemic therapy in patients with malignant disease” have been modified. In the long version of the guideline, new text passages and significant content‐related changes are marked in blue font color. For reasons of clarity, omitted text passages have not been highlighted.

*For the sections “Notes on using the guideline/Disclaimer” (these apply equally to the present short versions), “Funding”, “Scope and purpose of the guideline”, “Population and health questions covered by the guideline”, and “Targeted users of this guideline”, see the long version*.

## DISEASE SEVERITY AND TREATMENT GOALS

### Measuring disease activity

Although it has its drawbacks, the most established parameter to measure the severity of skin symptoms in patients with psoriasis is the *Psoriasis Area and Severity Index* (PASI), which was first introduced in 1978 as an outcome measure in a retinoid trial.^1^ A physician global assessment (PGA) score to evaluate disease severity can be beneficial in daily clinical care in order to rapidly assess the severity of psoriasis. It is important to note that different PGAs exist and that they may differ in the way they are defined and scales that are used. An estimate of the percentage of the affected body surface area (BSA) is also used as a means to measure disease severity.^2^ Health‐related quality of life (HRQoL) is another important aspect of psoriasis, not only in defining disease severity but also as an outcome measure in clinical trials. So far, the Dermatology Life Quality Index (DLQI) has been the most commonly used score for assessing the impact of psoriasis on HRQoL. However, the construct validity of the DLQI has been challenged: items answered as being “not relevant” by a specific patient are not always accompanied by a corresponding adjustment in the final result of the patient's DLQI.^3^ In addition to the DLQI, the *WHO‐5* is available, reflecting the concept of people‐centered healthcare.^4^ With the *ActiPso*, a tool is available defining the disease activity of psoriasis.^5^

### Defining disease severity

Defining disease severity in psoriasis is complex, and a multitude of clinical aspects and aspects related to HRQoL need to be taken into consideration. The currently available scores have various limitations and, as patients have repeatedly pointed out, none of the available scores can fully capture the complexity of the disease. As aptly put by Mara Maccarone and Ray Jobling, patient representatives of the *European Dermatology Forum* (EDF) *Guidelines* 2015: “*Severity has become defined technically and bureaucratically, in terms of scores derived from instruments like PASI, DLQI, and Skindex‐25. These simply fail to capture the seriousness of psoriasis as experienced by those who have the disease*.”

Currently, the disease definition most commonly used for psoriasis is strongly influenced by the definition used in clinical trials. It was thoroughly discussed and formally agreed upon in a European consensus project in 2011.

**Table:** 

ii.‐1 | modified [2025] **Definition of psoriasis disease severity** Mild psoriasis:^6^ BSA ≤ 10 and PASI ≤ 10 and DLQI ≤ 10 Moderate to severe psoriasis:^6^ (BSA > 10 or PASI > 10) and DLQI > 10 Criteria to further “upgrade” mild disease to moderate‐to‐severe:^6^ major involvement of visible areas, major involvement of the scalp, involvement of the genitals, palms of hands or soles of feet, onycholysis or onychodystrophy of at least two fingernails, pruritis leading to scratching, and presence of recalcitrant plaques.^6^ Criteria for particularly severe psoriasis include PASI ≥ 20, DLQI ≥ 15; rapid deterioration of findings, severe involvement of hands and/or feet, scalp, face, nails, or genitals.	**Statement**	strong consensus consensus‐based

### Treatment goals

The fundamental goal of any therapy is to achieve complete clearance of symptoms – that is, the absence of cutaneous symptoms of psoriasis. However, this goal is not realistically achievable in all patients at this time.

The successful establishment of treatment goals requires that a minimum target is defined which must be achieved by therapy. If this “lowest hurdle” is not reached within a given amount of time, the therapy must be modified. Various forms of adjustment include increasing the dosage, initiation of combination therapy, or transitioning to another drug or procedure. Treatment goals must be determined individually with the patients in a joint decision‐making process.

**Table:** 

iii.‐1 | reviewed [2025] At the end of the induction period, a PASI 75 response is the minimum target, which should be controlled for at regular intervals during the course of treatment.	**Statement**	strong consensus ^*^ consensus‐based
iii.‐2 | new [2025] Reaching a PASI 90 response or an absolute PASI <2 or DLQI ≤ 1 should be the desired treatment goal.	**↑↑**	strong consensus ^*^ consensus‐based
iii.‐3 | reviewed [2025] In the presence of criteria such as distinct involvement of visible areas, involvement of major parts of the scalp, genitals, palms of hands or feet, onycholysis or onychodystrophy of at least two fingernails, pruritus leading to scratching, or presence of recalcitrant plaques, we recommend to follow up treatment goals individually determined for this specific manifestation (using appropriate scores, e.g., *Nail Psoriasis Severity Index* [NAPSI]) and to modify the therapy, if necessary.	**Statement**	strong consensus ^*^ consensus‐based

*Five abstentions due to conflicts of interest.

For treatments with a fast onset of action, treatment goals should be controlled for at the end of the induction therapy after 10 to 12 weeks; for treatments with a slower onset of action, this should be done after 16 to 24 weeks. These time frames may not always include the time of maximum therapeutic effect. During maintenance therapy, control of treatment goals should be done at the same intervals as the general monitoring, usually every 8 to 12 weeks.

Another treatment goal that is gaining attention is the prevention of a chronic course of psoriasis. Studies are currently underway to investigate the potential impact of a particularly early initiation of treatment on disease chronicity.

For the section “Time to onset of action”: see the long version.

## METHODS

*For more detailed information, see the Guideline Development Report* (https://register.awmf.org/de/leitlinien/detail/013-001).

Standardized wording as suggested by the *GRADE Working Group* was used for all recommendations in the newly developed sections (Table 1).^7^

**TABLE 1:** Standardized recommendation formulations (adapted from^8^, ^9^, ^10^).

Strength	Wording	Symbols	Implications
Strong recommendation for the use of an intervention	“We recommend …”	**↑↑**	We believe that all or almost all informed people would make a choice in favor of using this intervention. Clinicians will not have to spend as much time on the process of decision‐making with the patients and may devote that time instead to overcoming barriers to implementation and adherence. In most clinical situations, the recommendation can be adopted as a policy.
Weak recommendation for the use of an intervention	“We suggest …”	**↑**	We believe that most informed people would make a choice in favor of using this intervention, but a substantial number would not. Clinicians and other health care providers will need to devote more time to the process of shared decision‐making. Decision processes in the health system will require substantial debate and involvement of many stakeholders.
Open recommendation**/**no recommendation	“We cannot make a recommendation for or against …”	0	Currently, a recommendation in favor of or against using this intervention cannot be made due to certain circumstances (for example, unclear or balanced benefit‐risk ratio, no data available, etc.)
*Weak* recommendation against the use of an intervention	“We suggest against …”	↓	We believe that most informed people would make a choice against using this intervention, but a substantial number would not.
*Strong* recommendation against the use of an intervention	"We recommend against …”	↓↓	We believe that all or almost all informed people would make a choice against using this intervention. In most clinical situations, the recommendation can be adopted as a policy.

Each formally agreed upon recommendation is presented in the guideline in a box: the left column shows the content of the recommendation using standardized wording or guideline language; the middle column shows the direction and the strength of the recommendation; and the right column indicates the strength of consensus among the expert group and the evidence base (expert consensus vs. evidence‐based).

*For the sections “Consensus procedure”, “External review”, “Approval by commissioning societies”, “Updates”, “Validity”, and “How to read and understand a network meta‐analysis” – see long version*.

## GENERAL RECOMMENDATIONS

### Initiating and selecting a systemic therapy

**Table:** 

ix‐1 | reviewed [2025] *We recommend* taking into account efficacy (see Figure 1, Figure 2, and summary of the Cochrane Review), safety (see drug chapters), comorbidities (see Table 1 and Table 2 in publication Part 2, and respective chapters in the long version), and individual patient factors when choosing a systemic treatment for moderate to severe psoriasis.	**↑↑**	strong consensus ^*^ evidence‐ and consensus‐based (see Evidence Report, chapter 1) CoE: see Figure 2 and Figure 3
ix‐2 | modified [2025] *We recommend* initiating a systemic treatment in patients with moderate to severe psoriasis according to Figure 3.	**↑↑**	strong consensus* evidence‐ and consensus‐based (see Evidence Report, chapter 1) CoE: see Figure 2 and Figure 3
ix‐3 | new [2025] If systemic treatment of psoriasis is initiated for the first time, *we suggest* treatment with a biologic with first‐line label or with deucravacitinib, especially for psoriasis of particular severity (see chapter “Defining disease severity”).	**↑**	strong consensus* evidence‐ and consensus‐based (see Evidence Report, chapter 1) CoE: see Figure 2 and Figure 3

* Abstentions due to conflicts of interest: 5.

**FIGURE 1:**
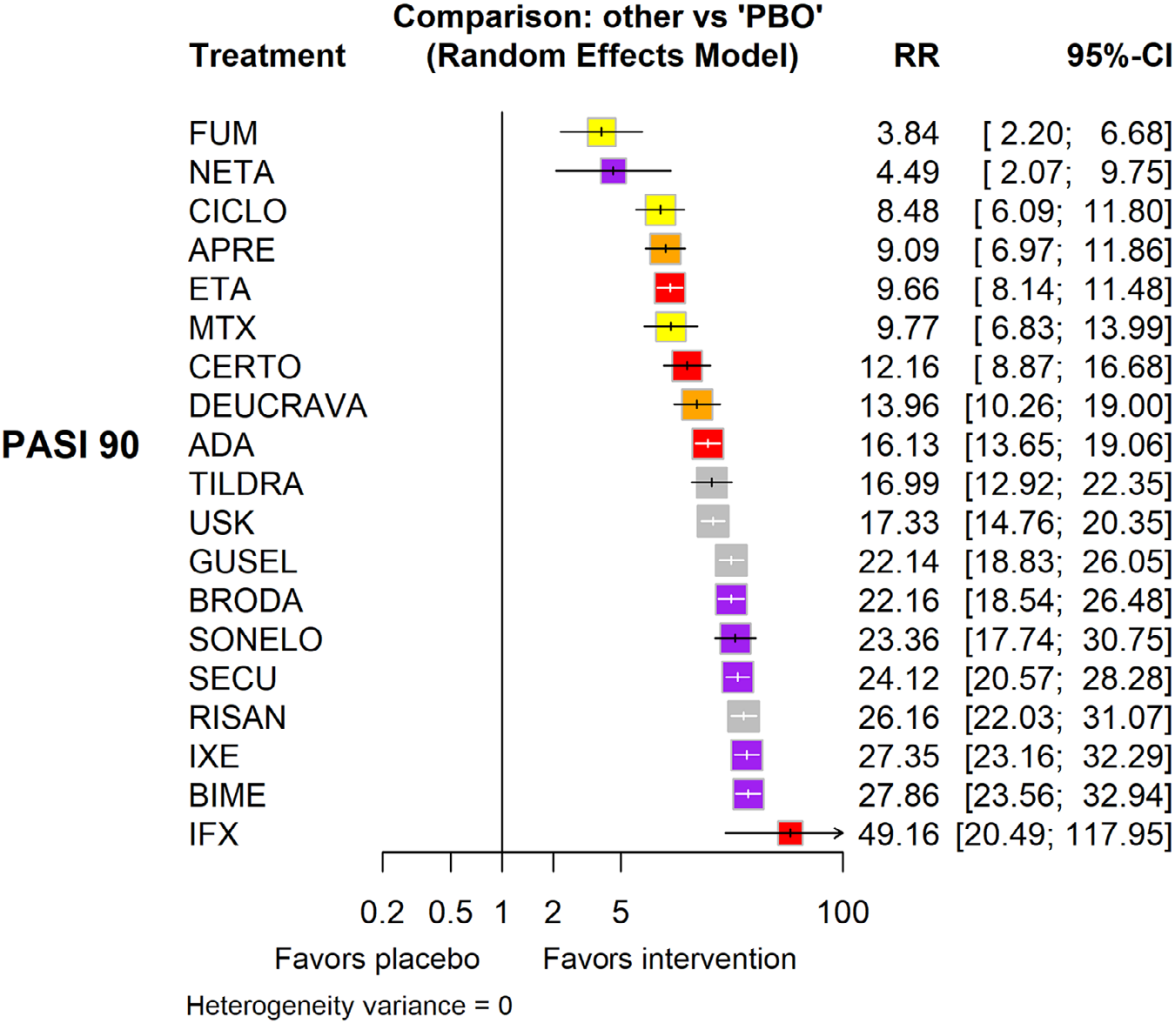
Forest Plot (relative effects versus placebo), reproduced from [Copyright © 2023 The Cochrane Collaboration].^11^

**FIGURE 2:**
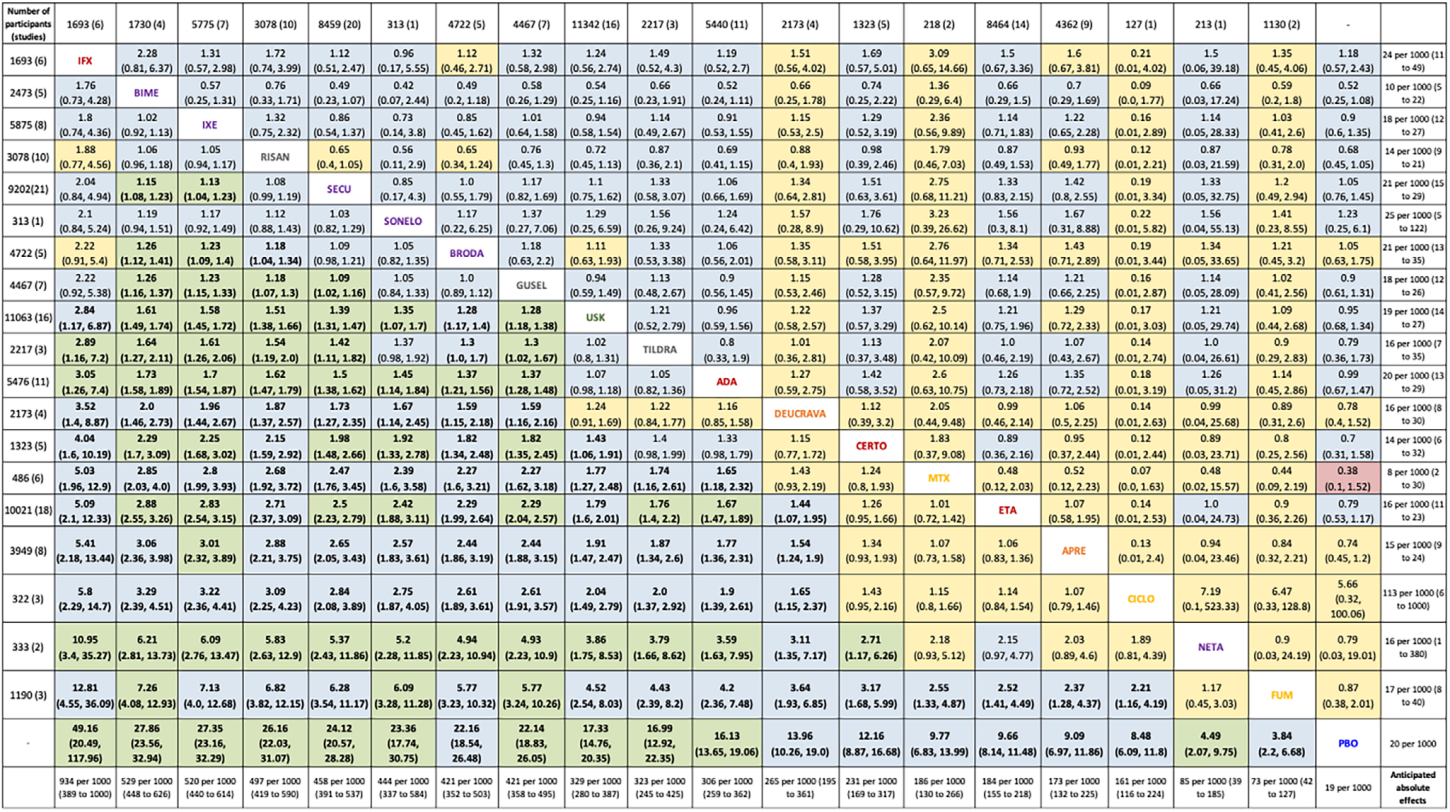
League table of relative effects (PASI 90: lower triangle; SAE: upper triangle), Certainty of Evidence (CINeMA): high (green), moderate (blue), low (yellow), very low (red). [Copyright © 2023 The Cochrane Collaboration].^11^

**FIGURE 3:**
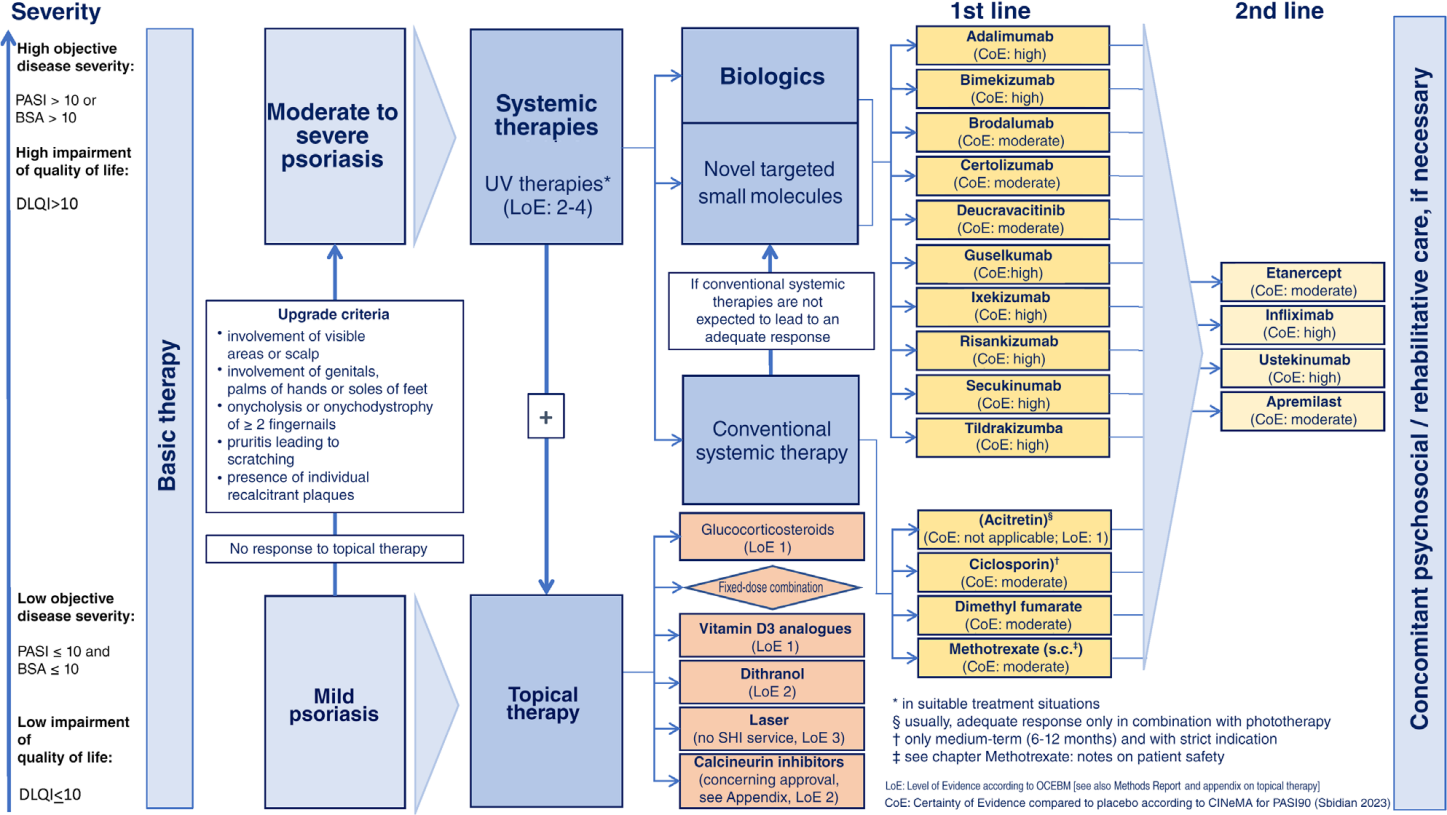
Overview of treatment options (CoE: refer to Figure 2 for network comparisons of PASI 90 and serious adverse events).

#### Evidence base

The systemic treatment options outlined in Figure 3 and the recommendations IX‐1 to IX‐3 are based on the results of the Cochrane review by Sbidian et al.^11^ Since the review demonstrated high methodological quality in the assessment using the AMSTAR II tool and is updated at regular intervals, no further systematic reviews on this topic were sought.

The statements on topical and UV therapies are based on the systematic evidence synthesis last updated in 2015. The results of this synthesis have been published as an appendix to the guideline.

#### Reasoning for Figure 3 and the associated recommendations IX‐1 to IX‐3

Figure 3 describes the treatment options in mild and moderate‐to‐severe psoriasis. The transition is defined by formally agreed upgrade criteria^6^ (see chapter II “Defining disease severity”).

Given that all systemic therapies studied in the Cochrane review^11^ showed a significant benefit compared to placebo with respect to the outcome measure PASI 90 (“*certainty of evidence*”: high – moderate) and the relative risk of SAEs did not differ significantly between the respective interventions and placebo, the guideline group gives a strong positive recommendation (IX‐2) for initiating a systemic therapy in patients with moderate to severe psoriasis.

Based on the results of the network comparisons for the outcome measures PASI 90 and SAE alone, the guideline group is not able to sufficiently differentiate the recommendation levels for the individual agents.

A wide range of treatment options are currently available, and the criteria for selecting a suitable agent are complex. The guideline group therefore considers it necessary to evaluate not only aspects of cost effectiveness, but also the individual wishes and characteristics of patients, for example, comorbidities and risks of infection, and to correlate these aspects with specific safety aspects of the individual agents (see recommendation IX‐1).

Accordingly, individual recommendation strengths arise individually from the examination of each patient, taking the factors effectiveness, safety, comorbidity, and other individual patient factors into account.

To support the assessment of suitability in case of comorbidity, the guideline group refers to the drug chapters (see section 0) highlighting particularly significant aspects of the summary of product characteristics, in addition to the narrative presentation of evidence in the following section.

Moreover, recommendations have been established in the chapters on special clinical and comorbidity situations (see section 3). These recommendations are illustrated In Table 1 and Table 2 in Part 2 of the publication and may support users of the guideline in decision‐making.

The recommendation IX‐3 on the direct use of first‐line biologics or deucravacitinib is justified by the particularly high disease burden in particularly severe psoriasis and based on the results of the Cochrane review^11^ (see Figure 2).

The classification of agents in 1st line and 2nd line, as presented in Figure 3, was based on the approved indications listed in the summary of product characteristics.

#### Results of the network meta‐analysis^11^

“[…] Network meta‐analysis^11^ at class level showed that all interventions (non‐biological systemic agents, small molecules, and biological treatments) showed a higher proportion of patients reaching PASI 90 than placebo.”^11^ (Figure 1, 2).

“Anti‐IL‐17 treatment showeda higher proportion of patients reaching PASI 90 compared to all other interventions.

Biologic treatments anti‐IL‐17, anti‐IL‐12/23, anti‐IL‐23, and anti‐TNF alpha showed a higher proportion of patients reaching PASI 90 than the non‐biologic systemic agents.

For reaching PASI 90, the most effective drugs when compared to placebo were (SUCRA rank order, all high certainty evidence):infliximab (risk ratio [RR] 49.16, 95% confidence interval [CI] 20.49–117.95), bimekizumab (RR 27.86, 95% CI 23.56–32.94), ixekizumab (RR 27.35, 95% CI 23.15–32.29), risankizumab (RR 26.16, 95% CI 22.03–31.07).

Clinical effectiveness of these drugs was similar when compared against each other. Bimekizumab and ixekizumab were significantly more likely to reachPASI 90 than secukinumab.

Bimekizumab, ixekizumab, and risankizumab were significantly more likely to reach PASI 90 than brodalumab and guselkumab. Infliximab, anti‐IL‐17 drugs (bimekizumab, ixekizumab, secukinumab, and brodalumab), and anti‐IL‐23 drugs except tildrakizumab were significantly more likely to reach PASI 90 than ustekinumab, three anti‐TNF alpha agents, and deucravacitinib. Ustekinumab was superior to certolizumab. Adalimumab, tildrakizumab, and ustekinumab were superior to etanercept. No significant difference was shown between apremilast and two non‐biologic drugs: ciclosporin and MTX.”^11^ (Figure 2).

“We found no significant difference between any of the interventions and the placebo for the risk of SAEs. The risk of SAEs was significantly lower for participants on methotrexate compared with most of the interventions.

Nevertheless, the SAE analyses were based on a very low number of events with very low‐ to moderate‐certainty evidence for all the comparisons. The findingd therefore have to be viewed with caution.

For other efficacy outcomes (PASI 75, PGA 0/1), the results were similar to the results for PASI 90. Information on quality of life was often poorly reported and was absent for several of the interventions […]”.^11^

With respect to the methods, it should be noted that the outcome measures were evaluated for a period of 8 to 24 weeks after randomization, that the main analysis – for which the *certainty of evidence* was assessed – included doses not approved by the European Medicines Agency (EMA), and that no clear differentiation between “therapy‐related” and “non‐therapy‐related” severe adverse events (SAEs) was performed.

#### Additional aspects on safety

Apart from the results on severe adverse events reported in the network meta‐analysis, the guideline group highlights notable safety aspects in the individual drug chapters.

#### Additional aspects on onset of action

The time until onset of action that is acceptable for patients is an individual factor. Depending on the patients’ symptoms a longer time until onset of action may be acceptable, if the effectiveness of the agent is ultimately satisfactory.

Various studies are currently investigating the possibility of modifying the disease course of psoriasis through early initiation of systemic therapy.^12^ The guideline group refers to the ongoing discussion and acknowledges a potential need to update the guideline in the future, including the impact on indication and selection of a systemic therapy, if applicable.

*For the sections “Recommendations for basic therapy”, “Topical therapy”, “Phototherapy”, and “Other therapies”, see document Appendix A (https://register.awmf.org/de/leitlinien/detail/013‐001)*.

## CONVENTIONAL SYSTEMIC THERAPY

*For sections “Acitretin”, “Ciclosporin”, and the complete chapter “Dimethyl fumarate”, see long version*.

### Dimethyl fumarate

In Europe, one DMF‐containing medication (Skilarence^®^) is approved and available for treating psoriasis. According to information from December 18, 2024, production of the fumaric acid ester Fumaderm^®^ has been discontinued in Germany.^13^

### Methotrexate (MTX)

Two meta‐analyses are available for comparison of the efficacy of oral versus subcutaneous administration of MTX in immune‐mediated diseases.^14^, ^15^ One meta‐analysis from 2019^14^ reported a higher efficacy for subcutaneous administration while no significant difference was found in the other meta‐analysis from 2022.^15^

With respect to toxicity, some, but not all, studies have shown that subcutaneous administration is better tolerated than oral administration.^16^

Pharmacokinetic studies indicate a higher plasma bioavailability and an increased accumulation of MTX polyglutamates (MTX‐PG) in red blood cells (RBC) for subcutaneous administration during the initial treatment phase. However, after several months similar intracellular drug levels for both administration pathways are observed.^17^ For doses above 15 mg, a better bioavailability was reported for subcutaneous administration.^18^

Especially, if doses above 15 mg are administered or if the risk of overdosing needs to be avoided, subcutaneous administration is preferred (because oral intake has a higher risk of overdosing as patients are more likely to take tablets daily instead of once weekly). The guideline group refers to the presence of a direct healthcare professional communication (Rote‐Hand‐Brief), emphasizing the risk of overdosing due to daily administration.^19^ The recommended initial and maintenance dose is usually 15 mg MTX once weekly. In case of insufficient response, the dose can be increased up to 20 mg MTX once weekly. A further increase up to 25 mg MTX is only beneficial for a small subgroup of patients. An increase beyond this dose is not recommended.^20^

#### Instructions for use MTX

**Table:** 

*Pre‐treatment*: Medical history and clinical examination Objective assessment of the severity of psoriasis (for example, using PASI/BSA/PGA; arthritis) Measure health‐related quality of life (for example, using DLQI/Skindex‐29 or ‐17) Laboratory monitoring (see Table 2) Exclusion of severe renal dysfunction (see chapter 3.6 “Kindney disease” in long version) Effective contraception in women of child‐bearing age (see chapter 3.11 “Wish for child/pregnancy” in long version) Concerning the requirement of protection against conception in male users of MTX, we refer to the chapter “Wish for child/pregnancy”: and to the valid version of the respective summary of product characteristics If abnormalities in liver screening are found, the patient should be referred to a specialist for further evaluation Check need for vaccinations (see chapter “Vaccinations”) *During treatment*: Medical history and clinical examination Objective assessment of the severity of psoriasis (for example, using PASI/BSA/PGA; arthritis) Measure health‐related quality of life (for example, using DLQI/Skindex‐29 or ‐17) Check concomitant medication Laboratory monitoring (see Table 2) Effective contraception in women of child‐bearing age (see chapter “Wish for child/pregnancy”) Concerning the requirement of protection against conception in male users of MTX, we refer to the chapter “Wish for child/pregnancy” in the long version and to the valid version of the respective summary of product characteristic 5 mg folic acid once weekly 24 hours after MTX Advise alcohol abstinence If respiratory tract symptoms are found (for example, suspected alveolitis), the patient should be referred to a specialist for further evaluation *Post‐treatment*: Concerning the requirement of protection against conception in female or male users beyond the time of MTX administration, we refer to the chapter “Wish for child/pregnancy” in the long version and to the valid version of the respective summary of product characteristics

modified [2025] | strong consensus; abstentions due to conflicts of interest: 5 | Follow‐up vote on renal dysfunction: strong consensus; abstentions due to conflicts of interest: 6.

#### Recommendations for laboratory monitoring

See table 2.

**TABLE 2:** Recommended Laboratory Monitoring for MTX.

Diagnostic workup	Time			
	*Pre‐treatment*	*Within the first 2 weeks*	*Within the first 3 months risk‐adapted, for example, in case of diabetes mellitus, obesity/ hepatic steatosis*	*Subsequently, every 3 months*
Blood count (including white blood cell differential)^*^	x	x	x	x
ALT, AST, gamma‐GT, AP^**^	x		x	x
Serum creatinine	x		x	x
Urinalysis	x			
Pregnancy test (urine or blood)	x			
Hepatitis B and hepatitis C serology	x			
HIV serology	x			
Serum albumin^***^	x		x	x

Not all tests may be required for all patients. Medical history, risk exposure, and patient characteristics have to be considered. Dependent on clinical signs, risk, and exposure, additional specific tests may be required.

* If leukocytes <3.0, neutrophils <1.0, thrombocytes <100, dose reduction or end of therapy

** If results of liver function tests > 2–3 x compared to baseline: initiate further diagnostic workup (repeat measurement/see respective specialist) and consider dose reduction or end of therapy

*** In selected cases (for example, suspected hypoalbuminemia, or if patients take other drugs with high binding affinity to serum albumin)

The recommendations regarding laboratory monitoring are based on clinical experience.

modified [2025] | strong consensus; abstentions due to conflicts of interest: 5

#### Adverse drug reactions

For more detailed information/complete presentation, see the summary of product characteristics and additional sources. The guideline group has decided to comment on the following aspects:

The most important adverse drug reactions (ADRs) in association with MTX therapy are mucositis, MTX‐induced pneumonitis, hepatotoxicity, and myelosuppression.

MTX‐induced pneumonitis may constitute a rare, but potentially life‐threatening hypersensitivity reaction. Therefore, patients need to be informed in detail about the requirement of making immediate contact in case of emerging dry cough with dyspnea without signs of upper respiratory tract infection (rhinitis, sinusitis, bronchitis).

The question of monitoring for the possible development of liver fibrosis is a recurring topic of intense debate.^21^ A potential overestimation of the role of MTX in development of liver fibrosis has been discussed in various publications, and the relevance of the frequent comorbidity of psoriasis with obesity and diabetes mellitus as more important factor has been emphasized.^22^, ^23^, ^24^, ^25^

Determination of transaminases for monitoring of potential liver damage due to MTX is the usual approach taken in the guideline group. If abnormalities are found, consultation of a corresponding specialist is recommended. Methods for further evaluation include calculation of the fibrosis‐4 (FIB‐4) index and elastography.^26^

Additional risk factors for hepatotoxicity include alcohol consumption, obesity (BMI ≥ 30), and inadequately controlled diabetes mellitus.^27^, ^28^ These three risk factors may result in hepatic steatosis with steatohepatitis and may thus increase MTX‐related hepatotoxicity.

Another rare, but serious adverse event of MTX therapy is myelosuppression with an estimated prevalence of <1% (7/100,000 patient years).^29^ This adverse event is usually the result of overdosing, for example, due to daily administration or due to an ignored or newly developed high‐grade renal insufficiency with creatinine clearance of <60 ml/min or <30 ml/min. Informing patients about the early symptoms of pancytopenia (sore throat, fever, stomatitis/problems of oral mucosa, and bleeding) may contribute to early detection.

#### Selection of important adverse drug reactions

**Table:** 

Very common	Nausea, malaise, hair loss
Common	Elevated transaminases, myelosuppression, gastrointestinal ulcers
Occasionally	Fever, chills, depression, infections
Rare	Nephrotoxicity, liver fibrosis, and liver cirrhosis
Very rare	Interstitial pneumonia and alveolitis

#### Special considerations during treatment

For more detailed information/complete presentation, see the summary of product characteristics and additional sources. The guideline group has decided to comment on the following aspects:

If gastrointestinal symptoms are experienced during MTX therapy, consumption of coffee and/or dark chocolate may be beneficial in up to 30% of patients.^30^

*Older patients*: Particular care is needed when treating geriatric patients where the dosage should generally be lower than usual. Moreover, kidney function should be monitored regularly in these patients. (CAUTION if creatinine clearance <60 ml/min, especially with concomitant diuretic therapy, medication with ACE inhibitors, warm weather conditions).

#### Important contraindications

For more detailed information/complete presentation, see the summary of product characteristics and additional sources. The guideline group has decided to comment on the following aspects:

*Absolute contraindications*

- Severe infections
- Severe liver diseases
- Severe renal dysfunction
- Active wish for child in women of child‐bearing age, pregnancy/breastfeeding (see chapter “Pregnancy” in the long version)
- Alcohol abuse
- Bone marrow failure/hematological abnormalities
- Acute gastric ulcer
- Significantly reduced pulmonary function
- Insufficient understanding of administration once weekly

*Relative contraindications*

- Kidney or liver diseases
- Old age
- Ulcerative colitis
- Gastritis
- Obesity (BMI > 30)
- Diabetes mellitus (uncontrolled, creatinine clearance <60 ml/min and albuminuria > 30 mg/g, creatinine in spot urine)^31^, ^32^
- Malignant diseases (see chapter “Cancer”)

#### Drug interactions

For more detailed information/complete presentation, see the summary of product characteristics and additional sources. The guideline group has decided to comment on the following aspects:

A number of drugs including salicylates, sulfonamides, diphenyl hydantoin, and several antibiotics (that is, penicillin, tetracyclines, chloramphenicol, trimethoprim) may reduce the binding of MTX to serum albumin, thus increasing the risk of MTX toxicity. Tubular secretion is inhibited by probenecid. Particular care is needed in patients concomitantly using azathioprine or retinoids. Some NSAIDs may increase MTX levels and thus also MTX toxicity. It is therefore recommended to administer NSAIDs at other times of day than MTX. Folic acid should be taken at an interval of 24 hours after MTX administration. Given that MTX is no longer detected in blood after 24 hours, no reduced effectiveness due to intake of folic acid is anticipated. There is some evidence that the combination of MTX and folic acid may reduce adverse reactions without impairing effectiveness.^33^, ^34^, ^35^

##### List of the most important drug interactions

**Table:** 

**Substances**	**Type of interaction**
Colchicine, ciclosporin (CsA), NSAIDs, penicillin, probenecid, salicylates, sulfonamides	Reduced renal elimination of MTX
Chloramphenicol, cotrimoxazole, cytostatic agents, ethanol, NSAIDs, pyrimethamine, sulfonamides	Increased risk of bone marrow and gastrointestinal toxicity
Barbiturates, cotrimoxazole, phenytoin, probenecid, NSAIDs, sulfonamides	Interaction with binding to plasma proteins
Ethanol, leflunomide, retinoids, tetracyclines	Increased hepatotoxicity

*Measures in case of overdosing: see long version*.

## THERAPY WITH BIOLOGICS AND NEW TARGETED SMALL MOLECULES

*For the sections “Adalimumab”, “Apremilast”, “Brodalumab”, “Certolizumab pegol”, “Etanercept”, “Guselkumab”, “Infliximab”, “Ixekizumab”, “Risankizumab”, “Secukinumab”, “Tildrakizumab”, “Ustekinumab”, and “Newly approved drugs and therapies in development”, see long version*.

### Bimekizumab

#### Instructions for use Bimekizumab

**Table:** 

*Pre‐treatment*: Medical history and clinical examination including prior exposure to treatments, malignancies, infections (for example, candidiasis), and inflammatory bowel disease Consider enrolling the patient in a psoriasis registry Objective assessment of the severity of psoriasis (for example, using PASI/BSA/PGA; arthritis) Measure health‐related quality of life (for example, using DLQI/Skindex‐29 or ‐17) Other recommended measures include: Exclusion of skin cancer Check for lymphadenopathy Laboratory monitoring (see Table 3) Exclusion of tuberculosis (see chapter “Tuberculosis”) Exclusion of active infection Check need for vaccinations Reliable contraception *During treatment*: Medical history and clinical examination with focus on infections (in particular upper respiratory tract, candida, tuberculosis), contraception, and symptoms of inflammatory bowel disease Objective assessment of the severity of psoriasis (for example, using PASI/BSA/PGA; arthritis) Measure health‐related quality of life (for example, using DLQI/Skindex‐29 or ‐17) Laboratory monitoring (see Table 3) *Post‐treatment*: After cessation of bimekizumab therapy: follow‐up with medical history and physical examination For information regarding the need for ongoing contraception immediately following biologic treatment cessation, see chapter “Wish for child/pregnancy”

new [2025] | consensus, abstentions due to conflicts of interest: 4.

#### Recommendations for laboratory monitoring

See table 3.

**TABLE 3:** Recommended Laboratory Monitoring for Bimekizumab.

Diagnostic workup	Time	
	*Pre‐treatment*	*Every 3–6 months*
Blood count (including white blood cell differential)	x	x
Liver function tests	x	x
Pregnancy test (urine or blood)	x	
Hepatitis B and hepatitis C serology	x	
HIV serology	x	
Interferon gamma release assay (IGRA) (exclusion of tuberculosis)	x	

Not all tests may be required for all patients. Medical history, risk exposure, and patient characteristics have to be considered. Dependent on clinical signs, risk, and exposure, additional specific tests may be required.

The recommendations regarding laboratory monitoring are based on clinical experience.

new [2025] | consensus; abstentions due to conflicts of interest: 5.

#### Adverse drug reactions

For more detailed information and a complete list, we refer to the summary of product characteristics and additional sources. The guideline group has decided to comment on the following aspects:

In the opinion of the guideline group, bimekizumab has, with the exception of candidiasis, a similar safety profile to other IL‐17 antagonists, such as ixekizumab and secukinumab, as well as the IL‐17R antagonist brodalumab.

In all phase III trials (BE READY^36^, BE VIVID^37^, BE SURE^38^, and BE RADIANT^39^), bimekizumab was well tolerated.

*Characteristics of the pooled analysis by Gordon et al.* *40*: Safety data were pooled from a cohort of patients from four randomized clinical phase II trials (BE ABLE 1, BE ABLE 2, PS0016, and PS0018) and four randomized clinical phase III trials (BE VIVID, BE READY, BE SURE, and BE BRIGHT).^40^ In this analysis, a total of 1,789 patients (1,252 [70.0%] male; mean age [SD], 45.2 [13.5] years) were treated with one or more doses of bimekizumab. The total exposure to bimekizumab was 3,109.7 person‐years.^40^ Treatment‐emergent adverse events (TEAEs) occurred with an exposure‐adjusted incidence rate (EAIR) of 202.4 per 100 person‐years and did not increase with increasing exposure to bimekizumab.^40^ EAIRs for suicidal ideation and behavior (0.0 per 100 person‐years; 95% CI 0.0–0.2 per 100 person‐years) and severe cardiovascular events (0.5 per 100 person‐years; 95% CI 0.3–0.8 per 100 person‐years) were low.^40^

*Neutropenia*: The EAIR for neutropenia was 0.8 per 100 person‐years (95% CI 0.6–1.2 per 100 person‐years).^40^

*Infections*: The three most common TEAEs were nasopharyngitis (19.1 per 100 person‐years; 95% CI 17.4–20.9 per 100 person‐years), oral candidiasis (12.6 per 100 person‐years; 95% CI 11.3–14.0 per 100 person‐years), and infections of the upper respiratory tract (8.9 per 100 person‐years; 95% CI 7.8–10.1 per 100 person‐years).^40^ Most cases of oral candidiasis were mild or moderate; three events resulted in treatment discontinuation.^40^

*Inflammatory bowel disease*: Limited data are available on patients with inflammatory bowel disease (IBD). In the pooled analysis by Gordon et al.^40^ described above, the EAIR for inflammatory bowel disease (0.1 per 100 person‐years; 95% CI 0.0–0.3 per 100 person‐years) was low. Patients with a known history of Crohn's disease were excluded from the clinical phase III trials. One case of ulcerative colitis on bimekizumab therapy was reported. It is recommended to exercise caution in patients with a history of IBD when prescribing bimekizumab.

*Candidiasis*: In addition to the results of the pooled analysis by Gordon et al.^40^ reported under the subheading “Infections”, the results of one phase III trial^39^ were also included. In the trial BE RADIANT^39^, bimekizumab and secukinumab were compared in adult patients with moderate to severe psoriasis. The prevalence of candida infections over a period of 0 to 48 weeks was higher in the patients treated with bimekizumab (n = 79/373 [21.2%]) than in those treated with secukinumab (n = 17/370 [4.6%]).^39^

Dual inhibition of IL‐17A and IL‐17F may impair the protective function of mucous membranes more strongly, thus increasing the risk of oral candidiasis. Early treatment of candida infections with topical or systemic therapy is recommended. For more detailed information on the treatment of candidiasis, we refer to the summary of product characteristics of antimycotic agents or to (international) guidelines^41^, ^42^, ^43^. In case of recurrent mycotic infections, a change in psoriasis therapy may be considered. It has to be taken into account, however, that clinically significant, severe infections will always present a contraindication for all biologics.

#### Special considerations during treatment

For more detailed information and a complete list, we refer to the summary of product characteristics and additional sources. The guideline group has decided to comment on the following aspects:

*Surgery*: Currently, no data are known to the guideline group concerning surgery in patients treated with bimekizumab. The decision to withhold bimekizumab prior to surgery should be based on individual factors, such as type and risk of the surgical intervention, patient characteristics, severity of psoriasis if treatment is discontinued etc. Consultation with the treating surgeon is recommended.

#### Important contraindications

For more detailed information and a complete list, we refer to the summary of product characteristics and additional sources. The guideline group has decided to comment on the following aspects:

*Absolute contraindications*:

- Clinically relevant active infections

*Relative contraindications*:

- Pregnancy or breastfeeding
- Chronic inflammatory bowel diseases

*For the sections “Drug interactions” and “Measures in case of overdosing”, see long version*.

### Deucravacitinib

#### Instructions for use Deucravacitinib

**Table:** 

*Pre‐treatment*: Medical history and clinical examination (especially, prior exposure to treatments, malignancies, signs and risks of infection Consider enrolling the patient in a psoriasis registry Objective assessment of the severity of psoriasis (for example, using PASI/BSA/PGA; arthritis) Measure health‐related quality of life (for example, using DLQI/Skindex‐29 or ‐17) Other recommended measures include: Exclusion of skin cancer Check for lymphadenopathy Exclusion of tuberculosis (see chapter “Tuberculosis”) Exclusion of active and chronic infections Check need for vaccinations according to current vaccination recommendations including prophylactic vaccination against herpes zoster Laboratory monitoring (see Table 4) Exclusion of pregnancy or breastfeeding Reliable contraception Inform patients to discontinue treatment and present for examination if they experience muscle weakness or pain, especially if these are accompanied by fatigue or fever. *During treatment*: Medical history and clinical examination, including assessment of risk factors for severe infections, signs of infection, and malignancies Objective assessment of the severity of psoriasis (for example, using PASI/BSA/PGA; arthritis) Measure health‐related quality of life (for example, using DLQI/Skindex‐29 or ‐17) Laboratory monitoring (see Table 4) Reliable contraception

**Table:** 

*Post‐treatment*: After cessation of treatment with deucravacitinib, follow‐up with medical history and physical examination For information regarding the need for ongoing contraception immediately following treatment cessation, see chapter “Wish for child/pregnancy”

new [2025] | strong consensus; abstentions due to conflicts of interest: 5.

#### Recommendations for laboratory monitoring

See table 4.

**TABLE 4:** Recommended Laboratory Monitoring for Deucravacitinib.

Diagnostic workup	Time	
	*Pre‐treatment*	*Only in case of corresponding history‐related or clinical indications*
Blood count (including white blood cell differential)	x	(x)
Liver function tests	x	(x)
Serum creatinine	x	(x)
Pregnancy test (urine or blood)	x	(x)
Hepatitis B and hepatitis C serology	x	(x)
HIV serology	x	(x)
Creatine kinase (CK)	x	(in case of muscle pain during treatment)
Interferon gamma release assay (IGRA) (exclusion of tuberculosis)	x	(x)

Not all tests may be required for all patients. Medical history, risk exposure, and patient characteristics have to be considered. Dependent on clinical signs, risk, and exposure, additional specific tests may be required.

The recommendations are based on expert opinions and take into account that the experience with the drug is still limited. The recommended laboratory controls exceed those suggested in the summary of product characteristics^44^ (as of July 2024).

new [2025] | consensus, abstentions due to conflicts of interest: 6.

#### Adverse drug reactions

For more detailed information/complete presentation, see the summary of product characteristics^44^ and additional sources.^45^, ^46^, ^47^, ^48^ The guideline group has decided to comment on the following aspects:

The most common adverse drug reactions (occurring in ≥ 1% and with a higher rate than in the placebo group) in the combined data from the trials POETYK PSO‐1 and POETYK PSO‐2 until week 16 were infections of the upper respiratory tract, elevated creatin kinase levels in blood, herpes simplex, oral ulcers, folliculitis, and acne. In addition, headache, diarrhea, and nausea were reported, all with similar frequency in deucravacitinib group and placebo group. Until week 52, no new adverse drug reactions were identified, and their incidence rates did not increase compared to those observed in the first 16 weeks of treatment.

##### Summary of Key Adverse Events (Deucravacitinib)

**Table:** 

Very common	Infections of upper respiratory tract^*^
Common	Herpes simplex infections^**^, oral ulcerations^***^, acneiform rash^****^, folliculitis
Occasionally	Herpes zoster

* Nasopharyngitis, infection of upper respiratory tract, viral infection of upper respiratory tract, pharyngitis, sinusitis, acute sinusitis, rhinitis, tonsillitis, peritonsillar abscess, laryngitis, tracheitis, and rhinotracheitis

** Cold sore, herpes simplex, genital herpes, and other herpes virus infections

*** Oral aphthae, ulcerations of oral mucosa, tongue ulcers, and stomatitis

**** Acne, acneiform dermatitis, exanthema, rosacea, pustules, papulopustular skin lesions

##### Infections

Deucravacitinib may increase the risk of infection. The majority of the observed infections were not severe, but mild to moderate, and included infections of the upper respiratory tract that did not result in treatment discontinuation. The most frequent severe infections reported with deucravacitinib treatment were pneumonias and COVID‐19 which can be attributed to the pandemic situation.

Reactivation of herpes viruses (for example, herpes zoster, herpes simplex) were reported in clinical trials. Most cases of herpes zoster were mild to moderate, restricted to one dermatome, had a mild course and did not result in cessation of treatment. During the pivotal trials POETYK PSO‐1, PSO‐2 and the open‐label extension study, ten of the 18 patients reporting herpes zoster were less than 50 years old. Furthermore, there was one case of herpes zoster with involvement of several dermatomes in an immunocompetent participant receiving deucravacitinib. Physicians should inform patients about early signs and symptoms of herpes zoster and advise them to initiate treatment as soon as possible.

##### Changes of laboratory findings

Pooled data from clinical trials on changes in laboratory findings show that treatment with deucravacitinib may result in the following changes in laboratory findings: increase of creatine kinase (from asymptomatic to rhabdomyolysis), increase of triglyceride levels and increase of liver enzymes by ≥ 3‐fold of the upper limit of normal. Deucravacitinib treatment should be interrupted if myopathy or liver damage is suspected. Patients should be instructed to present for examination if they experience muscle weakness or pain, especially if these are accompanied by fatigue or fever.

##### Malignancies

In the pooled data from the complete treatment periods of the pivotal trials PSO‐1, PSO‐2, and the open‐label extension study (overall, 2,482 patient‐years of exposure with deucravacitinib), malignancies were reported in 22 patients (0.9 per 100 patient‐years), including eleven cases of non‐melanoma skin cancer (0.4 per 100 patient‐years) and three cases of lymphoma (0.1 per 100 patient‐years).

#### Special considerations during treatment

For more detailed information/complete presentation, see the summary of product characteristics^44^ and additional sources.^47^, ^48^, ^49^, ^50^ The guideline group has decided to comment on the following aspects:

##### Potential risks in association with JAK inhibitors

Based on safety concerns, both the *U.S. Food and Drug Administration* (FDA) and EMA endorsed measures to minimize the risk of severe cardiovascular events, malignancies, thrombotic events, and death in association with Janus kinase (JAK) inhibitors.

It is not known whether deucravacitinib can be associated with the observed or potential adverse reactions of other JAK inhibitors. Deucravacitinib is a highly selective TYK2 inhibitor with minimal or no activity against JAK 1/2/3 in clinically relevant doses and concentrations. The allosteric mechanism of TYK2 inhibition reduces the risk of *off‐target* effects, and data from PSO‐1, PSO‐2, and the nonblinded extension study showed consistent safety profiles of deucravacitinib in patients with psoriasis. Nevertheless, further observations are required to completely characterize the long‐term safety of deucravacitinib.

##### Surgery

No data are available on the management of surgeries in patients treated with deucravacitinib. The decision to interrupt deucravacitinib treatment prior to surgery should be made on a case‐by‐case basis. Type and risk of the surgical intervention, patient characteristics, risk of infection, and risk of aggravation of psoriasis should be considered. Consultation with the surgeon is recommended.

The guideline of the *American College of Rheumatology/American Association of Hip and Knee Surgeons* for perioperative management of antirheumatic drugs in patients with rheumatic diseases undergoing elective total hip or knee arthroplasty recommends to withhold JAK inhibitors for at least 3 days prior to surgery.

#### Important contraindications

*For more detailed information/complete presentation, see the summary of product characteristics* *44* *and additional sources*.*45* *^–^* *47* *The guideline group decided to comment on the following aspects*:

*Absolute contraindications*

- Hypersensitivity to the active ingredient or one of the excipients
- Active tuberculosis or another active severe infection

*Relative contraindications*

- Severe liver disease
- Pregnancy

Risks and benefits of the treatment with deucravacitinib should be carefully considered before initiating the therapy in patients that have chronic or recurrent infections, are at risk of tuberculosis, have a history of severe or opportunistic infections, or have underlying diseases making them susceptible to infections.

#### Drug interactions

For more detailed information/complete presentation, see the summary of product characteristics^44^ and additional sources.^47^, ^51^ The guideline group has decided to comment on the following aspects:

According to the results from studies on heathy volunteers, there were no clinically significant differences in the pharmacokinetics of deucravacitinib, when it was administered together with drugs inhibiting or inducing various drug‐metabolizing enzymes and transporters. These included: ciclosporin (dual Pgp/BCRP inhibitor), fluvoxamine (CYP1A2 inhibitor), ritonavir (CYP1A2 inductor), diflunisal (UGT1A9 inhibitor), pyrimethamine (OCT1 inhibitor), famotidine (H2 receptor antagonist), or rabeprazole (proton pump inhibitor). No clinically significant differences in the pharmacokinetics of the following drugs were observed when administered together with deucravacitinib: rosuvastatin, methotrexate, mycophenolate mofetil, and oral contraceptives (norethindrone acetate and ethinylestradiol).

Combination therapy of deucravacitinib with other immunomodulatory agents, including biologics or phototherapy, has not been evaluated in plaque psoriasis.

#### Overdosing/measures in case of overdosing

“Deucravacitinib has been administered in healthy subjects as single doses up to 40 mg (> 6 times the recommended human dose of 6 mg/day) and in multiple doses up to 24 mg/day (12 mg twice daily) for 14 days without dose‐limiting toxicity.

In case of overdose, it is recommended that the patient be monitored for any signs or symptoms of adverse reactions and appropriate symptomatic treatment instituted immediately. Dialysis does not substantially clear deucravacitinib from systemic circulation […].”^44^

## BIOSIMILARS

At the time of preparing this guideline, biosimilars for adalimumab, etanercept, infliximab, and ustekinumab^52^ were available in Europe. The recommendations of this guideline apply equally to the original molecule and the corresponding biosimilars.

*For the section “Guidance for specific clinical and comorbid situations”, see part 2 in the next issue of JDDG*.

## Note on Guideline adaptation

The authors of this work have adapted, remixed, transformed, translated or built upon a previous version of the following article: EUROGUIDERM GUIDELINE FOR THE SYSTEMIC TREATMENT OF PSORIASIS by Nast A et al.; which is available in its final form on the European Dermatology Forum website (https://www.guidelines.edf.one/guidelines/psoriasis-guideline) (licensed under CC BY NC 4.0, https://creativecommons.org/licenses/by-nc/4.0/):

– A Nast, PI Spuls, C Dressler, Z Bata‐Csörgö, I Bogdanov, H Boonen, EMGJ De Jong, I Garcia‐Doval, P Gisondi, D Kaur‐Knudsen, S Mahil, T Mälkönen, JT Maul, S Mburu, L Mercieca, U Mrowietz, A Pennitz, E Remenyik, D Rigopoulos, PG Sator, M Schmitt‐Egenolf, M Sikora, K Strömer, O Sundnes, G Van Der Kraaij, N Yawalkar, C Zeyen, C Smith. EUROGUIDERM GUIDELINE FOR THE SYSTEMIC TREATMENT OF PSORIASIS VULGARIS September 2023, partial update February 2025.

Furthermore, this article is based on an adaptation of the previous version of the German version of the guideline, which has been published in its final form at https://doi.org/10.1111/ddg.14508 and https://doi.org/10.1111/ddg.14507:

– Nast A, Altenburg A, Augustin M, Boehncke WH, Härle P, Klaus J, Koza J, Mrowietz U, Ockenfels HM, Philipp S, Reich K, Rosenbach T, Schlaeger M, Schmid‐Ott G, Sebastian M, von Kiedrowski R, Weberschock T, Dressler C. German S3‐Guideline on the treatment of Psoriasis vulgaris, adapted from EuroGuiDerm – Part 1: Treatment goals and treatment recommendations. J Dtsch Dermatol Ges. 2021 Jun;19(6):934‐150. doi: 10.1111/ddg.14508.
– Nast A, Altenburg A, Augustin M, Boehncke WH, Härle P, Klaus J, Koza J, Mrowietz U, Ockenfels HM, Philipp S, Reich K, Rosenbach T, Schlaeger M, Schmid‐Ott G, Sebastian M, von Kiedrowski R, Weberschock T, Dressler C. German S3‐Guideline on the treatment of Psoriasis vulgaris, adapted from EuroGuiDerm – Part 2: Treatment monitoring and specific clinical or comorbid situations. J Dtsch Dermatol Ges. 2021 Jul;19(7):1092‐1115. doi: 10.1111/ddg.14507.

The present adapted guideline did not undergo an approval procedure by the European Dermatology Forum, but has been approved by the editing German societies. This guideline is subject to the provisions of Creative Commons Attribution NonCommercial license.

## CONFLICT OF INTEREST STATEMENT

For the authors of the German version, see Guideline Development Report of the German adaptation at https://register.awmf.org/de/leitlinien/detail/013‐001. For the authors of the EuroGuiDerm version, see Methods Report: https://www.guidelines.edf.one/guidelines/psoriasis‐guideline [last accessed April 10, 2025].

## References

[1] Fredriksson T , Pettersson U . Severe psoriasis – oral therapy with a new retinoid. Dermatologica. 1978;157:238‐244. 357213 10.1159/000250839

[2] Puzenat E , Bronsard V , Prey S , et al. What are the best outcome measures for assessing plaque psoriasis severity? A systematic review of the literature. J Eur Acad Dermatol Venereol. 2010;24(Suppl 2):10‐16. 10.1111/j.1468-3083.2009.03562.x 20443995

[3] Rencz F , Brodszky V , Gulácsi L , et al. Time to revise the Dermatology Life Quality Index scoring in psoriasis treatment guidelines. J Eur Acad Dermatol Venereol. 2019;33:e267‐e269. 30821001 10.1111/jdv.15537

[4] World Health Organization . WHO‐Five well‐being index. 1998. Available from: https://www.psykiatri‐regionh.dk/who‐5/Pages/default.aspx [Last accessed June 27, 2024].

[5] Mrowietz U , Dieckmann T , Gerdes S , et al. ActiPso: definition of activity types for psoriatic disease: A novel marker for an advanced disease classification. J Eur Acad Dermatol Venereol. 2021;35:2027‐2033. 34076926 10.1111/jdv.17434

[6] Mrowietz U , Kragballe K , Reich K , et al. Definition of treatment goals for moderate to severe psoriasis: a European consensus. Arch Dermatol Res. 2011;303:1‐10. 20857129 10.1007/s00403-010-1080-1 PMC3016217

[7] Guyatt G , Oxman AD , Akl EA , et al. GRADE guidelines: 1. Introduction‐GRADE evidence profiles and summary of findings tables. J Clin Epidemiol. 2011;64:383‐394. 21195583 10.1016/j.jclinepi.2010.04.026

[8] Guyatt GH , Oxman AD , Schunemann HJ , et al. GRADE guidelines: a new series of articles in the Journal of Clinical Epidemiology. J Clin Epidemiol. 2011;64:380‐382. 21185693 10.1016/j.jclinepi.2010.09.011

[9] The GRADE Working Group . Available from: http://www.gradeworkinggroup.org/ [Last accessed July 10, 2025].

[10] Werner RN , Nikkels AF , Marinovic B , et al. European consensus‐based (S2k) Guideline on the Management of Herpes Zoster – guided by the European Dermatology Forum (EDF) in cooperation with the European Academy of Dermatology and Venereology (EADV), Part 1: Diagnosis. J Eur Acad Dermatol Venereol. 2017;31:9‐19. 27804172 10.1111/jdv.13995

[11] Sbidian E , Chaimani A , Guelimi R , et al. Systemic pharmacological treatments for chronic plaque psoriasis: a network meta‐analysis. Cochrane Database Syst Rev. 2023. 10.1002/14651858.CD011535.pub6 PMC10337265 37436070

[12] Lwin SM , Azrielant S , He J , Griffiths CEM . Curing Psoriasis. J Invest Dermatol. 2024;144:2645‐2649. 39436345 10.1016/j.jid.2024.09.012

[13] Biogen GmbH . Informationsschreiben des Unternehmens Biogen GmbH zur Einstellung der Produktion und des Vertriebs von Fumaderm® initial und Fumaderm® (Wirkstoffe: Dimethylfumarat/Ethylhydrogenfumarat). 2024. Available from: https://www.bfarm.de/SharedDocs/Arzneimittelzulassung/Lieferengpaesse/DE/2024/info_Dimethylfumarat_Ethylhydrogenfumarat_10241206.pdf?__blob=publicationFile [Last accessed December 18, 2024].

[14] Bujor AM , Janjua S , LaValley MP , et al. Comparison of oral versus parenteral methotrexate in the treatment of rheumatoid arthritis: A meta‐analysis. PLoS One. 2019;14:e0221823. 31490947 10.1371/journal.pone.0221823 PMC6731021

[15] Wang F , Tang J , Li Z , et al. Oral methotrexate at doses 15‐25 mg/week is non‐inferior to parenteral regarding efficacy and safety in the treatment of rheumatoid arthritis: a systematic review and meta‐analysis. Clin Rheumatol. 2022;41:2701‐2712. 35672619 10.1007/s10067-022-06221-z

[16] Vermeer E , Hebing RCF , van de Meeberg MM , et al. Oral Versus Subcutaneous Methotrexate in Immune‐Mediated Inflammatory Disorders: an Update of the Current Literature. Curr Rheumatol Rep. 2023;25:276‐284. 37768405 10.1007/s11926-023-01116-7 PMC10754736

[17] Dalrymple JM , Stamp LK , O'Donnell JL , et al. Pharmacokinetics of oral methotrexate in patients with rheumatoid arthritis. Arthritis Rheum. 2008;58:3299‐3308. 18975321 10.1002/art.24034

[18] Schiff MH , Jaffe JS , Freundlich B . Head‐to‐head, randomised, crossover study of oral versus subcutaneous methotrexate in patients with rheumatoid arthritis: drug‐exposure limitations of oral methotrexate at doses ≥15 mg may be overcome with subcutaneous administration. Ann Rheum Dis. 2014;73:1549‐1551. 24728329 10.1136/annrheumdis-2014-205228 PMC4112421

[19] Arzneimittelkommission der deutschen Ärzteschaft (AkdÄ) . Methotrexat: Maßnahmen zur Vermeidung von Dosierungsfehlern mit potenziell tödlichen Folgen bei der Anwendung von Methotrexat bei Autoimmunerkrankungen. Rote‐Hand‐Brief vom 25.11.2019. 2019. Available from: https://www.akdae.de/fileadmin/user_upload/akdae/Arzneimittelsicherheit/RHB/Archiv/2019/20191125.pdf [Last accessed May 06, 2024].

[20] van Huizen AM , Menting SP , Gyulai R , et al. International eDelphi Study to Reach Consensus on the Methotrexate Dosing Regimen in Patients With Psoriasis. JAMA Dermatol. 2022;158:561‐572. 35353175 10.1001/jamadermatol.2022.0434

[21] Barker J , Horn EJ , Lebwohl M , et al. Assessment and management of methotrexate hepatotoxicity in psoriasis patients: report from a consensus conference to evaluate current practice and identify key questions toward optimizing methotrexate use in the clinic. J Eur Acad Dermatol Venereol. 2011;25:758‐764. 21198946 10.1111/j.1468-3083.2010.03932.x

[22] Atallah E , Grove JI , Crooks C , et al. Risk of liver fibrosis associated with long‐term methotrexate therapy may be overestimated. J Hepatol. 2023;78:989‐997. 36702175 10.1016/j.jhep.2022.12.034

[23] Bafna P , Sahoo RR , Hazarika K , et al. Prevalence of liver fibrosis by Fibroscan in patients on long‐term methotrexate therapy for rheumatoid arthritis. Clin Rheumatol. 2021;40:3605‐3613. 33686476 10.1007/s10067-021-05678-8

[24] Slouma M , Lahmar W , Mohamed G , et al. Associated factors with liver fibrosis in rheumatoid arthritis patients treated with methotrexate. Clin Rheumatol. 2024;43:929‐938. 38159207 10.1007/s10067-023-06847-7

[25] Darabian S , Wade JP , Kur J , et al. Using FibroScan to Assess for the Development of Liver Fibrosis in Patients With Arthritis on Methotrexate: A Single‐center Experience. J Rheumatol. 2022;49:558‐565. 35293340 10.3899/jrheum.211281

[26] Roeb E , Canbay A , Bantel H , et al. Aktualisierte S2k‐Leitlinie nicht‐alkoholische Fettlebererkrankung der Deutschen Gesellschaft für Gastroenterologie, Verdauungs und Stoffwechselkrankheiten (DGVS). Z Gastroenterol. 2022;60:1346‐1421. 36100202 10.1055/a-1880-2283

[27] Bichenapally S , Khachatryan V , Muazzam A , et al. Risk of Liver Fibrosis in Methotrexate‐Treated Patients: A Systematic Review. Cureus. 2022;14:e30910. 36465792 10.7759/cureus.30910 PMC9711916

[28] Castiella A , Lopez‐Dominguez L , Sanchez‐Iturri MJ , et al. Liver steatosis in patients with rheumatoid arthritis treated with methotrexate is associated with body mass index. World J Hepatol. 2023;15:699‐706. 37305368 10.4254/wjh.v15.i5.699 PMC10251276

[29] Gutierrez‐Ureña S , Molina JF , García CO , et al. Pancytopenia secondary to methotrexate therapy in rheumatoid arthritis. Arthritis Rheum. 1996;39:272‐276. 8849378 10.1002/art.1780390214

[30] Malaviya AN . Methotrexate intolerance in the treatment of rheumatoid arthritis (RA): effect of adding caffeine to the management regimen. Clin Rheumatol. 2017;36:279‐285. 27596742 10.1007/s10067-016-3398-3

[31] Levey AS , de Jong PE , Coresh J , et al. The definition, classification, and prognosis of chronic kidney disease: a KDIGO Controversies Conference report. Kidney Int. 2011;80:17‐28. 21150873 10.1038/ki.2010.483

[32] Corrections to „The definition, classification, and prognosis of chronic kidney disease: a KDIGO Controversies Conference report“. Kidney Int. 2011;80:1000. 10.1038/ki.2011.310 30036909

[33] Duhra P . Treatment of gastrointestinal symptoms associated with methotrexate therapy for psoriasis. J Am Acad Dermatol. 1993;28:466‐469. 8445064 10.1016/0190-9622(93)70069-6

[34] Ortiz Z , Shea B , Suarez Almazor M , et al. Folic acid and folinic acid for reducing side effects in patients receiving methotrexate for rheumatoid arthritis. Cochrane Database Syst Rev. 2000;CD000951. 10796393 10.1002/14651858.CD000951

[35] van Ede AE , Laan RF , Rood MJ , et al. Effect of folic or folinic acid supplementation on the toxicity and efficacy of methotrexate in rheumatoid arthritis: a forty‐eight week, multicenter, randomized, double‐blind, placebo‐controlled study. Arthritis Rheum. 2001;44:1515‐1524. 11465701 10.1002/1529-0131(200107)44:7<1515::AID-ART273>3.0.CO;2-7

[36] Gordon KB , Foley P , Krueger JG , et al. Bimekizumab efficacy and safety in moderate to severe plaque psoriasis (BE READY): a multicentre, double‐blind, placebo‐controlled, randomised withdrawal phase 3 trial. Lancet. 2021;397:475‐486. 33549192 10.1016/S0140-6736(21)00126-4

[37] Reich K , Papp KA , Blauvelt A , et al. Bimekizumab versus ustekinumab for the treatment of moderate to severe plaque psoriasis (BE VIVID): efficacy and safety from a 52‐week, multicentre, double‐blind, active comparator and placebo controlled phase 3 trial. Lancet. 2021;397:487‐498. 33549193 10.1016/S0140-6736(21)00125-2

[38] Warren RB , Blauvelt A , Bagel J , et al. Bimekizumab versus Adalimumab in Plaque Psoriasis. N Engl J Med. 2021;385:130‐141. 33891379 10.1056/NEJMoa2102388

[39] Reich K , Warren RB , Lebwohl M , et al. Bimekizumab versus Secukinumab in Plaque Psoriasis. N Engl J Med. 2021;385:142‐152. 33891380 10.1056/NEJMoa2102383

[40] Gordon KB , Langley RG , Warren RB , et al. Bimekizumab Safety in Patients With Moderate to Severe Plaque Psoriasis: Pooled Results From Phase 2 and Phase 3 Randomized Clinical Trials. JAMA Dermatol. 2022;158:735‐744. 35544084 10.1001/jamadermatol.2022.1185 PMC9096693

[41] Pappas PG , Kauffman CA , Andes DR , et al. Clinical Practice Guideline for the Management of Candidiasis: 2016 Update by the Infectious Diseases Society of America. Clin Infect Dis. 2016;62:e1‐50. 26679628 10.1093/cid/civ933 PMC4725385

[42] Edwards SK , Bunker CB , van der Snoek EM , van der Meijden WI . 2022 European guideline for the management of balanoposthitis. J Eur Acad Dermatol Venereol. 2023;37:1104‐1117. 36942977 10.1111/jdv.18954

[43] Groll AH , Buchheidt D , Heinz W , et al. S1‐Leitlinie Diagnose und Therapie von Candida Infektionen: Gemeinsame Empfehlungen der Deutschsprachigen Mykologischen Gesellschaft (DMykG) und der Paul‐Ehrlich‐Gesellschaft für Chemotherapie (PEG) 2020. Available from: https://register.awmf.org/de/leitlinien/detail/082‐005 [Last accessed January 03, 2025].

[44] European Medicines Agency . Sotyktu: EPAR – Product Information. Available from: https://www.ema.europa.eu/en/medicines/human/EPAR/sotyktu [Last accessed September 26, 2023].

[45] Armstrong AW , Gooderham M , Warren RB , et al. Deucravacitinib versus placebo and apremilast in moderate to severe plaque psoriasis: Efficacy and safety results from the 52‐week, randomized, double‐blinded, placebo‐controlled phase 3 POETYK PSO‐1 trial. J Am Acad Dermatol. 2023;88:29‐39. 35820547 10.1016/j.jaad.2022.07.002

[46] Strober B , Thaçi D , Sofen H , et al. Deucravacitinib versus placebo and apremilast in moderate to severe plaque psoriasis: Efficacy and safety results from the 52‐week, randomized, double‐blinded, phase 3 Program for Evaluation of TYK2 inhibitor psoriasis second trial. J Am Acad Dermatol. 2023;88:40‐51. 36115523 10.1016/j.jaad.2022.08.061

[47] Bristol Myers Squibb . SOTYKTU™ (deucravacitinib) tablets, for oral use: US prescribing information. 2022. Available from: https://packageinserts.bms.com/pi/pi_sotyktu.pdf [Last accessed February 01, 2023].

[48] Warren RB , Sofen H , Imafuku S . Deucravacitinib long‐term efficacy and safety in plaque psoriasis: 2‐year results from the phase 3 POETYK PSO program. presented at: Presented at European Academy of Dermatology and Venereology (EADV) Spring Symposium; May 12–14, 2022, 2022; Ljubljana, Slovenia.

[49] Chimalakonda A , Burke J , Cheng L , et al. Selectivity Profile of the Tyrosine Kinase 2 Inhibitor Deucravacitinib Compared with Janus Kinase 1/2/3 Inhibitors. Dermatol Ther (Heidelb). 2021;11:1763‐1776. 34471993 10.1007/s13555-021-00596-8 PMC8484413

[50] Goodman SM , Springer BD , Chen AF , et al. 2022 American College of Rheumatology/American Association of Hip and Knee Surgeons Guideline for the Perioperative Management of Antirheumatic Medication in Patients With Rheumatic Diseases Undergoing Elective Total Hip or Total Knee Arthroplasty. Arthritis Care Res (Hoboken). 2022;74:1399‐1408. 35718887 10.1002/acr.24893

[51] Catlett IM , Aras U , Hansen L , et al. First‐in‐human study of deucravacitinib: A selective, potent, allosteric small‐molecule inhibitor of tyrosine kinase 2. Clin Transl Sci. 2023;16:151‐164. 36325947 10.1111/cts.13435 PMC9841305

[52] European Medicines Agency . Uzpruvo: EPAR – Product Information. 2024. Available from: https://www.ema.europa.eu/en/medicines/human/EPAR/uzpruvo [Last accessed June 25, 2024].

